# Risk Factors Associated with Anterior Cruciate Ligament Injuries in Athletes During Physical Activity According to Sex: A Scoping Review

**DOI:** 10.3390/sports14060243

**Published:** 2026-06-12

**Authors:** Paula A. Rodríguez-Molina, Rafael Barrera, Laura S. Gonzalez, Juan G. Ortiz, Eduardo Tuta-Quintero

**Affiliations:** 1 Department of Orthopedics and Traumatology, Universidad de La Sabana, Chía 250001, Colombia; paularomo@unisabana.edu.co (P.A.R.-M.); rafaelbame@unisabana.edu.co (R.B.); lauragoncab@unisabana.edu.co (L.S.G.); 2 Department of Epidemiology and Internal Medicine, Universidad de La Sabana, Chía 250001, Colombia; eduardotuqu@unisabana.edu.co

**Keywords:** anterior cruciate ligament, non-contact injury, risk factors, sex differences, sports activity

## Abstract

**Background**: Anterior cruciate ligament (ACL) injury is one of the most common injuries among athletes and demonstrates significant sex-based differences in incidence, with a higher documented risk in females. Various anatomical, biomechanical, neuromuscular, and hormonal factors have been proposed to explain this disparity; however, the available evidence remains inconclusive due to methodological heterogeneity across studies, variability in outcome measures, and inconsistencies in the assessment of hormonal and biomechanical variables. **Objective**: To map and synthesize the scientific evidence regarding risk factors associated with ACL injury during sports activity, incorporating a sex-specific analytical perspective. **Methods**: A scoping review was conducted following the methodological framework proposed by Arksey and O’Malley, the Joanna Briggs Institute guidelines, and the PRISMA Extension for Scoping Reviews (PRISMA-ScR). A systematic search was performed in PubMed and Scopus through September 2025. Observational and experimental studies assessing ACL injury risk factors and analyzing sex-based differences were included. Findings were synthesized using a descriptive and narrative approach. **Results**: Nineteen studies were included. Biomechanical and neuromuscular factors were the most frequently investigated domains among the included studies (68.4%), followed by hormonal (42%) and anatomical factors (36.8%). These percentages reflect the distribution of research focus across the literature rather than the relative strength or importance of each risk factor. In females, injury risk was primarily associated with high-risk biomechanical patterns, cyclical hormonal variations, and specific anatomical characteristics. In males, risk factors were mainly related to muscular weakness, joint laxity, and structural ligament characteristics. **Conclusions**: ACL injury risk in athletes appears to be influenced by multiple interacting intrinsic and extrinsic factors. The available evidence suggests that sex-related differences may exist in the contribution of biomechanical, anatomical, hormonal, and neuromuscular factors; however, these relationships are multifactorial and should be interpreted cautiously given the heterogeneity of the included studies.

## 1. Introduction

Anterior cruciate ligament (ACL) injury is one of the most common injuries among athletes, particularly in sports that involve jumping, rapid changes in direction, and sudden decelerations [1]. In addition to resulting in prolonged periods of sports inactivity, ACL injury is associated with knee instability, secondary meniscal injuries, and an increased risk of early-onset osteoarthritis, representing a substantial burden on both athlete health and competitive performance [1]. The incidence of ACL injury differs according to sex [2], with females demonstrating a higher risk compared to males, with rates reported to be two to six times greater, particularly in sports such as soccer and basketball [2,3]. These differences have persisted over time despite the implementation of prevention programs. However, this persistence may reflect not only the multifactorial nature of ACL injury but also factors related to variability in program implementation, adherence levels among athletes, and differences in intervention design and real-world application [3].

Risk factors associated with the higher frequency of ACL injuries in females include anatomical, biomechanical, neuromuscular, and hormonal components [4]. Anatomical factors include differences in knee morphology and ligament size, whereas biomechanical and neuromuscular factors encompass movement patterns characterized by greater dynamic knee valgus and altered muscle activation during physical activity [4,5]. Hormonal factors have also been proposed as an additional determinant of ACL injury risk; however, the available evidence remains heterogeneous due to differences in study populations, variability in the definition of menstrual cycle phases, diverse hormone measurement methods, and inconsistencies in the outcomes evaluated across studies [6].

Cyclical fluctuations in estrogen, progesterone, and relaxin throughout the menstrual cycle have been suggested to influence ligament laxity, collagen composition, and neuromuscular control, thereby modifying the mechanical properties of the ligament during physical activity [7]. Observational studies have reported a higher incidence of non-contact injuries during the preovulatory or periovulatory phases, periods characterized by elevated estrogen concentrations [7,8,9]. At the tissue level, experimental research has demonstrated that relaxin may increase the expression of matrix metalloproteinases and reduce collagen synthesis in female ACL cells, suggesting a potential biological mechanism capable of compromising the structural integrity of the ligament [10]. However, the evidence remains heterogeneous due to methodological differences in defining menstrual cycle phases, hormone measurement techniques, and evaluated outcomes, which have limited the ability to establish definitive conclusions regarding the precise role of hormonal factors in ACL injury [11].

Observational studies indicate that female athletes experience ACL injuries approximately 2 to 6 times more frequently than male athletes in sports involving cutting and landings [7,8,9,10,11]. These findings suggest that anatomical, biomechanical, neuromuscular, and hormonal factors may interact differently between sexes [10,11]. Therefore, the risk of ACL injury during athletic activity is influenced by a multifactorial interaction of intrinsic and extrinsic factors with sex-specific characteristics [4,5,10,11]. Despite the growing body of literature, most existing reviews have primarily focused on specific domains, particularly biomechanical and neuromuscular risk factors, often analyzing these determinants separately. Consequently, a comprehensive synthesis that integrates anatomical, biomechanical, neuromuscular, and hormonal risk factors from a sex-specific perspective during sports activity remains limited [11]. In this context, a scoping review represents an appropriate methodological strategy to systematically map the available evidence, identify knowledge gaps, and guide future research and preventive strategies incorporating a sex-specific approach.

## 2. Methods

A scoping review was conducted following the methodological framework proposed by Arksey and O’Malley [12], subsequently refined by Levac et al. [13], as well as the methodological guidance provided by the Joanna Briggs Institute [14] for scoping reviews. Reporting was performed in accordance with the PRISMA Extension for Scoping Reviews (PRISMA-ScR) [15].

The review was carried out through a systematic five-stage process: (1) formulation of the research question, (2) identification of relevant studies, (3) study selection based on predefined criteria, (4) standardized data extraction, and (5) synthesis and presentation of findings.

### 2.1. Research Question

The research question was formulated using a modified PICOS framework to guide the search strategy and eligibility criteria: Population (athletes), Exposure (risk factors), Comparison (sex differences), Outcome (ACL injury), and Study design (observational and experimental studies) [16,17,18,19,20,21,22,23]. Accordingly, the research question guiding this review was, What sex-specific risk factors are associated with the occurrence of anterior cruciate ligament injuries during sports activity according to the available scientific literature? Given the multifactorial nature of ACL injury, a broad research question was intentionally adopted to facilitate the identification and mapping of the different domains of risk factors reported in the literature.

### 2.2. Eligibility Criteria

Studies conducted in athlete populations that evaluated risk factors associated with anterior cruciate ligament (ACL) injury, including anatomical, biomechanical, neuromuscular, and/or hormonal factors, and that analyzed sex differences or included populations allowing sex-based comparison were eligible for inclusion. Experimental and observational studies published in English or Spanish were considered. This language restriction was applied due to feasibility considerations during the screening and data extraction processes and may have limited the inclusion of relevant studies published in other languages.

Narrative reviews, editorials, letters to the editor, study protocols, and studies without full-text access were excluded (Table 1).

### 2.3. Information Sources and Search Strategy

Systematic searches were conducted in PubMed and Scopus (Table 2). The search strategy was developed using MeSH terms and keywords combined with Boolean operators. Studies published up to September 2025 were included.

### 2.4. Study Selection Process

Retrieved records were exported in RIS format, and duplicates were removed using reference management software and manual verification. Studies were subsequently imported into the Rayyan platform [16], where two independent reviewers screened titles and abstracts according to the predefined eligibility criteria.

Studies classified as uncertain or with discrepancies between reviewers were reassessed, and if disagreement persisted, a third reviewer determined inclusion or exclusion. Selected articles were then evaluated in full text following the same procedure. The entire process was documented using a PRISMA-ScR flow diagram [15].

### 2.5. Data Extraction and Synthesis

Data extraction was performed independently by two reviewers using a standardized template that included: author, year, study design, population characteristics, risk factors assessed, evaluation methods, and key findings.

Results were synthesized using a descriptive and narrative approach. Evidence was organized according to the type of risk factor evaluated (anatomical, biomechanical/neuromuscular, hormonal, and contextual), highlighting observed sex-based differences in alignment with the objectives of the review and the findings reported in the included studies.

## 3. Results

**Figure 1:**
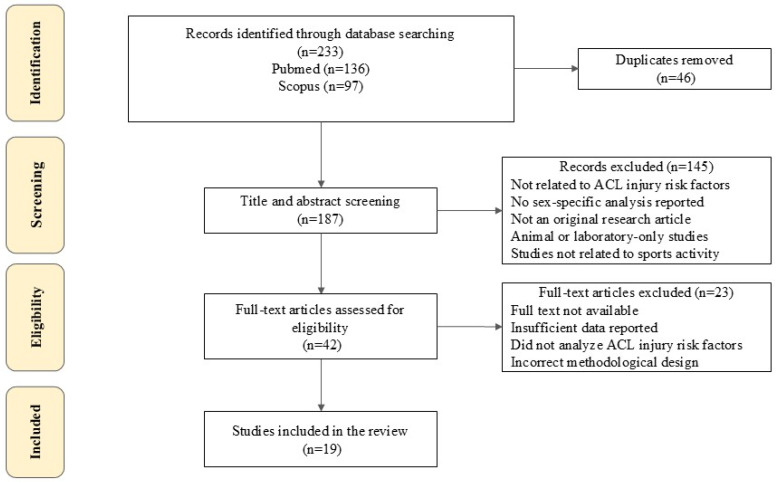
PRISMA Flow Diagram of Study Selection.

A total of 233 records were identified through systematic searches in PubMed (n = 136) and Scopus (n = 97) (Figure 1). After the selection process, 19 studies were included in the final synthesis.

Among the included studies, 26.3% (5/19) were prospective observational designs, 21.1% (4/19) were retrospective observational studies, and 15.8% (3/19) were cross-sectional studies. Additionally, 26.3% (5/19) employed a case–control design, while 10.5% (2/19) were experimental studies conducted in laboratory or cellular settings (Table 3).

Regarding the study population, 52.6% (10/19) included exclusively female participants, 10.5% (2/19) evaluated only male participants, and 36.8% (7/19) included both sexes, allowing for direct comparisons.

With respect to the risk factors assessed, biomechanical and neuromuscular factors were the most frequently analyzed, present in 68.4% (13/19) of the studies, primarily through motion analysis during functional tasks such as landing, jumping, and cutting maneuvers. Anatomical factors were evaluated in 36.8% (7/19) of studies, mainly using magnetic resonance imaging (MRI) to assess bony and ligamentous geometry. Hormonal factors were examined in 42% (8/19), predominantly in female populations, considering menstrual cycle phases, serum or salivary hormone concentrations, and cellular mechanisms. External or contextual factors were assessed in only 5% (1/19) of the studies.

The most used assessment methods included three-dimensional biomechanical analysis in 47.4% (9/19) of studies, knee MRI in 31.6% (6/19), standardized functional tests such as the drop vertical jump in 31.6% (6/19), joint laxity measurements using arthrometers in 21.1% (4/19), and hormonal or cellular analyses in 42.1% (8/19).

Overall, 57.9% (11/19) of the studies analyzed risk factors using a sex-specific approach. In females, findings were primarily associated with specific anatomical characteristics, high-risk biomechanical patterns, and cyclical hormonal variations. For example, Barnum et al. [31] identified that in female athletes, non-contact ACL injury was associated with a greater lateral tibial slope and a narrower intercondylar notch width, whereas these factors were not predominant in males. Similarly, Beaulieu et al. [32] reported that in females, ACL injury risk was associated with a greater lateral tibial cartilage slope and a reduced anterior intercondylar notch width, whereas different anatomical factors were observed in males.

From a biomechanical perspective, Peebles et al. reported that females demonstrated greater knee abduction angles and higher adduction moments during landing tasks, movement patterns that have been linked to an increased risk of non-contact ACL injury [18]. Likewise, Prados-Barbero et al., in elite judokas, found that females exhibited greater hip internal rotation, a factor that may influence knee mechanics during high-risk sporting maneuvers [19].

Regarding hormonal factors, Konopka et al. demonstrated at the cellular level that relaxin increased matrix metalloproteinase expression and reduced collagen and α-SMA synthesis in female ACL cells, suggesting a specific biological mechanism that could compromise ligament integrity in women [10].

In contrast, in males, risk factors were primarily associated with structural ligament characteristics, muscular weakness, and increased joint laxity. Dauty et al. reported that in male athletes, non-contact ACL injury was associated with older age, greater passive knee extension, and hamstring weakness, whereas these factors were not determinants in females [21].

## 4. Discussion

The present scoping review aimed to map and synthesize the available scientific evidence on risk factors associated with ACL injury during sports activity, incorporating a sex-specific perspective. The reviewed evidence indicates that ACL injury results from the interaction of multiple intrinsic and extrinsic factors, with a higher incidence observed in females [22,23]. The risk factors described in this review do not act independently but interact dynamically to influence susceptibility to ACL injuries [29,30,31,32,33,34,35,36]. A narrower intercondylar notch or greater tibial tilt can predispose the knee joint to altered load distribution, while neuromuscular deficits and biomechanical patterns, such as dynamic knee valgus, further amplify ligament stress during high-demand tasks [25,26,27,28,29,30,31,32,33,34,35,36]. Hormonal fluctuations can additionally modulate ligament laxity and neuromuscular activation, potentially exacerbating these biomechanical patterns during specific phases of the menstrual cycle [30]. This multifactorial interaction highlights the importance of considering ACL injury risk through a comprehensive model [31,32,33].

Among intrinsic factors, neuromuscular and biomechanical components emerge as some of the most relevant determinants. Several studies indicate that females exhibit reduced dynamic knee stability and less efficient muscle activation patterns during high-impact sports tasks, which increases joint loading during maneuvers such as landing and cutting [23,24]. These alterations manifest as landing patterns characterized by greater dynamic knee valgus and diminished neuromuscular control, which have been associated with an increased risk of injury in sports such as soccer, basketball, and volleyball [24,25]. These findings are consistent with previous biomechanical research indicating that increased knee valgus moments and altered neuromuscular control during landing tasks are key predictors of non-contact ACL injury, particularly in female athletes [24,30]. This supports the hypothesis that sex-related neuromuscular strategies during high-impact tasks may partially explain the higher incidence of ACL injuries observed in female populations.

Regarding hormonal factors, cyclical variations in estrogen and progesterone throughout the menstrual cycle have been proposed to influence knee laxity and stiffness, potentially altering ligament stability and neuromuscular control [26,27]. Specifically, some studies have reported reductions in knee joint stiffness during the ovulatory phase of the menstrual cycle, potentially associated with hormonal fluctuations that may influence ligament laxity and neuromuscular control [28]. However, these findings depend on the population studied, the measurement methods used, and considerable inter-individual variability, and therefore should be interpreted with caution [28,29,30,31,32,33,34,35].

Concerning anatomical factors, the literature describes morphological differences in knee structure that influence load distribution during movement and may contribute to increased injury risk, particularly when combined with reduced functional stiffness of the lower extremity [22]. In this context, Myer et al. demonstrated that biomechanical variables such as knee valgus, incorporated into a predictive algorithm, can identify athletes at high risk for ACL injury [24]. Similar findings were reported by Dauty et al., who observed that passive valgus was associated with non-contact ACL injury in females [21]. Additionally, Miljk et al. reported that the inner angle of the lateral femoral condyle represents a more robust predictor of ACL injury than intercondylar notch width [29].

With respect to extrinsic factors, these include variables related to the type of sport practiced, training intensity and experience, and sport-specific execution techniques, particularly during landing and cutting maneuvers. Sports involving jumping and sudden deceleration exhibit a higher incidence of ACL injury [24,30,31]. These findings have important clinical implications, as they support the implementation of neuromuscular training–based prevention programs, which have demonstrated significant reductions in modifiable risk factors [25,30,31]. From a clinical and preventive standpoint, the results facilitate the identification of individual risk profiles, enabling the development of personalized interventions aimed at optimizing hamstring strength, improving landing techniques, and reducing dynamic knee valgus during sports participation [24].

Current evidence supports the implementation of neuromuscular training programs focused on improving lower-limb alignment [30,32]. It is important to note that sex-specific considerations can further optimize the effectiveness of such interventions, especially in female athletes who have a higher incidence of injuries. Programs incorporating plyometric training, balance exercises, and technical correction during landing and cutting maneuvers have demonstrated significant reductions in the risk of ACL injury and should be systematically integrated into athletes’ development pathways [33,34,35,36].

These findings highlight the importance of implementing sex-specific prevention strategies in sports training programs [25,26,27,28,29,30,31,32,33,34,35]. Neuromuscular training interventions focused on improving landing mechanics, strengthening the hamstrings and hip stabilizers, and enhancing proprioceptive control have proven effective in reducing the risk of ACL injury in female athletes [33,34,35,36]. Furthermore, screening protocols that assess dynamic knee valgus, lower-limb strength asymmetries, and neuromuscular control can facilitate the early identification of athletes at higher risk and support the development of individualized injury prevention programs.

### Strengths and Limitations

The primary strength of this review lies in its comprehensive approach, which enabled the joint analysis of ACL injury risk factors and provided a broad perspective on the mechanisms involved. Furthermore, the sex-specific analysis represents a meaningful contribution by enhancing understanding of anatomical, biological, and functional differences that may inform individualized prevention and clinical management strategies.

Among the limitations, it should be noted that, as a scoping review, no formal methodological quality assessment of the included studies was conducted, which may affect the robustness of certain conclusions. Additionally, the heterogeneity of the analyzed populations—including athletes from different sports disciplines and competitive levels—limits direct comparability across studies. Another limitation is that the literature search was restricted to the Scopus and PubMed databases. Although these databases are widely recognized and comprehensive, the exclusion of other databases, such as Web of Science, may have resulted in the omission of some relevant studies.

External factors were assessed in only 5% (1/19) of the included studies, indicating a clear imbalance in the literature toward intrinsic determinants, such as biomechanical, neuromuscular, anatomical, and hormonal variables [29]. This limited representation of extrinsic factors may restrict the overall interpretation of ACL injury risk, as variables such as training load, sport-specific demands, playing surface, and environmental conditions may also influence injury incidence, yet they have been scarcely explored in the available evidence.

A strength of this review is its prospective registration on OSF (https://osf.io/tswy6; accessed on 17 March 2026), enhancing transparency and credibility. Limitations include heterogeneity of evidence and reliance on published data [37]. Future research should incorporate more rigorous methodological assessments, analyze more homogeneous populations, and further explore the influence of genetic and environmental factors among athletes at different competitive levels. Such advances would contribute to optimizing prevention, strengthening, and follow-up programs tailored to individual risk profiles.

## 5. Conclusions

ACL injury risk in athletes appears to be multifactorial and influenced by interacting biomechanical, neuromuscular, and anatomical factors in both sexes, with hormonal influences primarily investigated in female populations. The available evidence suggests substantial overlap in underlying mechanisms, and apparent sex-related differences may largely reflect differences in research focus rather than true biological distinctions. Further well-designed comparative studies are needed to clarify potential sex-specific contributions.

## Figures and Tables

**Table 1:** Database search strategies.

Search Strategies	
Database	Health Sciences Descriptors (DeCS) and Medical Subject Headings (MeSH) Terms Used
PubMed/MEDLINE	((“Anterior Cruciate Ligament Injuries”[Mesh] OR “Anterior Cruciate Ligament Reconstruction”[Mesh] OR “ACL injury” OR “ACL tear” OR “ACL rupture”) AND (“Risk Factors”[Mesh] OR “etiology”[Subheading] OR “risk factor” OR “predisposing factor” OR determinant) AND (“Sex Characteristics”[Mesh] OR “Sex Factors”[Mesh] OR “sex difference” OR “gender difference” OR “female athlete” OR “male athlete”) AND (“Athletes”[Mesh] OR athlete OR “elite athlete” OR “high-performance athlete” OR “competitive sport”) AND (“neuromuscular control” OR “landing mechanics” OR “jumping” OR “muscle imbalance” OR “menstrual cycle” OR estrogen OR progesterone OR “Q angle” OR “notch width” OR “joint laxity” OR “posterior tibial slope”)) Filters: database inception to 21 November 2025
Scopus	(TITLE-ABS-KEY(“ACL injury” OR “anterior cruciate ligament” OR “ACL tear” OR “ACL rupture”) AND TITLE-ABS-KEY(“risk factor” OR etiology OR determinant) AND TITLE-ABS-KEY(“sex difference” OR “gender difference” OR “female athlete” OR “male athlete”) AND TITLE-ABS-KEY(athlete OR “elite athlete” OR “high-performance athlete” OR “competitive sport”) AND TITLE-ABS-KEY(“neuromuscular control” OR “landing mechanics” OR “muscle imbalance” OR “menstrual cycle” OR estrogen OR progesterone OR “Q angle” OR “notch width” OR “joint laxity” OR “posterior tibial slope”))

**Table 2:** Inclusion and exclusion criteria.

Inclusion and Exclusion Criteria	
Inclusion Criteria	Exclusion Criteria
Studies conducted in athlete populations; Evaluation of risk factors associated with ACL injury; Risk factors: anatomical, biomechanical, neuromuscular, hormonal; Studies including sex-based analysis or allowing sex comparison; Study designs: Observational (prospective, retrospective, cross-sectional), case–control, and experimental studies; published in English or Spanish; Full-text available.	Narrative or systematic reviews; Editorials, letters to the editor, commentaries; Study protocols; Studies without analysis of risk factors; Studies without sex-differentiated data; Abstract-only publications; Duplicate publications.

**Table 3:** General characteristics of the study populations.

Author/Year	Study Design/Population	Risk Factor(s) Assessed	Assessment Method	Key Findings (Summary)
**Beynnon et al., 2006 [9]**	Case–control study; female skiers	Hormonal and sports experience	Blood analysis + questionnaire	Increased ACL rupture risk during preovulatory phase of the menstrual cycle.
**Konopka et al., 2016 [10]**	Experimental cellular study; ACL cells (M/F)	Hormonal and cellular	Cell culture and RT-PCR	Relaxin increased MMP expression and reduced collagen synthesis in female cells, suggesting a sex-specific hormonal mechanism.
**Peebles et al., 2020 [18]**	Experimental cross-sectional study; recreational athletes (M/F)	Biomechanical	3D analysis during DVJ and stop jump	Females demonstrated greater knee abduction and loading patterns associated with higher ACL injury risk.
**Prados-Barbero et al., 2024 [19]**	Cross-sectional study; male and female judokas	Biomechanical and neuromuscular	Single-leg squat and goniometry	Females showed greater hip internal rotation, potentially associated with increased ACL injury risk.
**Dauty et al., 2022 [21]**	Cross-sectional study; post-ACL athletes (M/F)	Anatomical and neuromuscular	Goniometry, KT-1000 arthrometer, dynamometry	In females, injury associated with passive valgus; in males, associated with older age, greater passive extension, and hamstring weakness.
**Anderson et al., 2001 [22]**	Prospective study; 100 basketball players (M/F)	Anthropometric and muscular strength	Anthropometry, dynamometry, MRI	Sex differences in body fat, muscle strength, and ACL size explain higher risk in females.
**Myer et al., 2012 [24]**	Retrospective + prospective validation; 744 female athletes	Biomechanical, neuromuscular, anthropometric	3D/2D analysis, DVJ, dynamometry	Clinical algorithm identified high-risk athletes with good sensitivity and specificity.
**Butler et al., 2013 [25]**	Prospective experimental study; 28 soccer players (M/F)	Sagittal-plane biomechanics	3D biomechanical analysis	Females exhibited lower lower-extremity stiffness, increasing passive loading and potential ACL injury risk.
**Wojtys et al., 1998 [26]**	Analytical observational study; 28 women with non-contact ACL injury	Hormonal (menstrual cycle)	Clinical questionnaire	Non-contact injuries were associated with specific menstrual cycle phases; limited by absence of objective hormonal confirmation.
**Yu et al., 1999 [27]**	Experimental cellular study	Hormonal (estrogen)	Cell culture and Western blot	Estrogen reduced fibroblast proliferation and collagen synthesis, suggesting decreased ligament strength.
**Park et al., 2009 [28]**	Case–control study; 26 healthy women	Hormonal and passive laxity	Serum hormones + KT-2000 arthrometer	Ovulation associated with increased laxity and decreased knee stiffness linked to hormonal variations.
**Miljko et al., 2012 [29]**	Prospective study; 51 female handball players	Anatomical, biomechanical, neuromuscular, and external factors	Orthopedic evaluation + knee MRI	Injury associated with a narrower intercondylar notch and greater lateral femoral condyle inner angle; the latter was the strongest predictor of ACL injury.
**Hewett et al., 2016 [30]**	Controlled clinical study; 467 young athletes	Biomechanical and neuromuscular	3D motion analysis and physical testing	High-risk biomechanical profiles were identified; individualized screening recommended for injury prevention.
**Barnum et al., 2021 [31]**	Case–control study; high school/collegiate athletes	Anatomical	Knee MRI	In females, injury associated with greater lateral tibial slope and narrower notch; in males, smaller ACL volume.
**Beaulieu et al., 2021 [32]**	Case–control study; high school/collegiate athletes	Anatomical	Knee MRI	In females, risk associated with greater lateral tibial slope and narrower notch; in males, smaller ACL volume.
**Park et al., 2009 [33]**	Controlled clinical trial; 26 healthy women	Biomechanical factors and knee laxity	Knee joint laxity (KJL) measurement and phase-based analysis	No biomechanical changes between phases; increased laxity associated with joint loading was observed.
**Stijak et al., 2015 [34]**	Case–control study; 58 young men	Hormonal and joint laxity	Serial salivary analysis and laxity scale	High testosterone levels associated with increased joint laxity and potential ACL injury risk.
**Liederbach et al., 2008 [35]**	Experimental study; 80 dancers/athletes	Biomechanical and neuromuscular	Single-leg landing + 3D analysis	Fatigue increased high-risk patterns; females demonstrated riskier mechanics regardless of fatigue.
**Adachi et al., 2008 [36]**	Retrospective observational study; 18 young women	Hormonal and clinical	Validated menstrual questionnaire	Higher injury frequency during ovulatory phase; activity level and premenstrual symptoms were not influential.

Notes: M/F: Male/female; ACL: anterior cruciate ligament; MRI: magnetic resonance imaging; DVJ: drop vertical jump; KJL: knee joint laxity; MMP: matrix metalloproteinases; α-SMA: alpha-smooth muscle actin.

## Data Availability

All data analyzed in this study are derived from previously published articles, which are cited within the manuscript.
